# Psoriasis seems often underdiagnosed in patient with axial spondyloarthritis

**DOI:** 10.1186/s13075-023-03119-2

**Published:** 2023-08-09

**Authors:** Angelique Rondags, Laura van Marle, Barbara Horváth, Freke R. Wink, Suzanne Arends, Anneke Spoorenberg

**Affiliations:** 1 Department of Dermatology, University of Groningen, University Medical Center Groningen, Groningen, The Netherlands; 2 Department of Rheumatology and Clinical Immunology, University of Groningen, University Medical Center Groningen, Groningen, The Netherlands; 3 Department of Rheumatology, Medical Center Leeuwarden, Leeuwarden, The Netherlands

**Keywords:** Axial spondyloarthritis, Psoriasis, Self-report, Prevalence, Surveys and questionnaires

## Abstract

**Background:**

Axial spondyloarthritis (axSpA) is known to be associated with several extra-skeletal manifestations (ESM), including the inflammatory skin disease psoriasis. It is important to recognize and diagnose psoriasis timely in axSpA in order to provide optimal treatment and outcome for both axSpA and psoriasis.

**Methods:**

In this observational study, all patients from the Dutch Groningen Leeuwarden Axial Spondyloarthritis (GLAS) cohort included before June 2016 were sent a questionnaire with self-screening psoriasis questions including prototypical color pictures.

**Results:**

Of the 592 questionnaires sent, 448 (75.7%) were eligible for analysis. Of these 448 respondents, 58 (13%) had a positive self-screening for psoriasis symptoms, currently or in the past. In 28 (48%) of 58 patients, psoriasis diagnosis could be verified by medical records, resulting in a psoriasis prevalence rate of 6.3%. In comparison with patients with a confirmed psoriasis diagnosis, patients reporting psoriasis symptoms without a verified diagnosis mentioned more mild than moderate-severe psoriasis symptoms (25% vs. 3%, *p* = 0.02), and their psoriasis lesions were less often located on the torso area (3% vs. 18%, *p* = 0.04), the intergluteal cleft (0% vs. 25%, *p* = 0.02), and legs (7% vs. 43%, *p* < 0.01). Of the 31 axSpA patients who reported currently active psoriasis, 74% had only mild psoriasis symptoms.

**Conclusions:**

Especially mild psoriasis seems often underdiagnosed in patients with axSpA using a patient questionnaire with prototypical pictures of psoriasis lesions. This questionnaire could be beneficial in tracing patients with undiagnosed psoriasis in daily clinical practice. As a next step, further validation of this questionnaire is needed.

## Key messages

- Psoriasis seems to be underdiagnosed in axSpA patients, although psoriasis, which is an extra-skeletal manifestation (ESM) of axSpA, may contribute to the diagnosis and to the pharmacological treatment decision of axSpA.
- A patient self-reported questionnaire with prototypical pictures to recognize psoriasis symptoms could be beneficial in tracing undiagnosed psoriasis.

## Introduction

Prior research has indicated that several extra-skeletal manifestations (ESM), including psoriasis, commonly occur in axial spondyloarthritis (axSpA) and are therefore part of the Assessment of Spondyloarthritis International Society (ASAS) criteria for axSpA since 2009 [1]. Besides psoriasis, ESM include inflammatory bowel diseases (IBD) such as Crohn’s disease, ulcerative colitis or undifferentiated colitis, and anterior uveitis.

Psoriasis is one of the most prevalent chronic inflammatory skin diseases, with a worldwide prevalence ranging from 0.1% in east Asia to 2.0% in Australia. For Western Europe, including the Netherlands, this prevalence is nearly 2%, with no clear differences between men and women [2]. Prevalence rates of psoriasis in axSpA vary between 3.1 and 18.9%, depending on the geographical area and inclusion criteria of the axSpA cohorts, with a pooled prevalence of around 10% and no significant difference between the axSpA subtypes: ankylosing spondylitis (AS) and non-radiographic (nr)-axSpA [3].

Psoriasis mostly presents as sharply demarcated, erythematous, silver-white, frequently coarse scaling papules and/or plaques, most commonly seen symmetrical on the elbows and knees (plaque-type psoriasis). This type is most reported in spondyloarthritis (SpA) [4]. However, other locations can be involved such as the scalp (psoriasis capitis), body folds (psoriasis inversa), palms, and soles which frequently include pustules (psoriasis pustulosa palmoplantaris) and nails (nail psoriasis). The diagnosis is usually made based on the typical clinical findings. If the dermatologist doubts the diagnosis, a skin biopsy can provide confirmation of the diagnosis. Treatment modalities vary based on location and the extent of psoriasis. These include topical therapies vitamin D analogs, corticosteroids, calcineurin inhibitors, and dithranol; phototherapy with ultraviolet A or B radiation; and systemic treatment such as methotrexate, fumarates, acitretin, and biological agents [5].

In the pathogenesis of both axSpA and psoriasis, deregulation of classic inflammatory cytokines such as interleukin (IL) 1, IL-12/23, IL-17, and IL-23 and tumor necrosis factor alpha (TNFα) are observed. However, how the immunological pathways including genetic background interact in patients with both axSpA and psoriasis still needs to be unraveled [6]. When a patient suffers from both active psoriasis and axial inflammation, systemic treatment usually concerns biologics instead of the aforementioned systemic treatment modalities, since these are not effective in axial inflammation. TNFα inhibitors or IL-17 antagonists are often the first choice since they have proven to be effective in both diseases [7].

Although axSpA and psoriasis are associated, in daily clinical practice awareness among treating physicians can still improve. Most rheumatologists ask for possible psoriatic skin involvement in patients suspected of axSpA during the first outpatient visit. However, during follow-up, they are not always aware of developing lesions and might have difficulty recognizing more atypical lesions [8]. Additionally, previous research has also shown that a significant proportion of patients with psoriasis may not seek medical attention and are unaware of the diagnosis [9]. Alike, the average diagnostic delay between the onset of axSpA symptoms and the axSpA diagnosis is 5–7 years [10]. Since psoriasis is part of the SpA features, it will contribute to adequate referral of patients suspected of axSpA and the SpA diagnosis in general. Furthermore, awareness of treating physicians, general practitioners, and patients that axSpA and psoriasis are associated also helps to provide for the most optimal treatment choices for both diseases. Importantly, patient self-recognition of signs and symptoms of psoriasis will help self-care including earlier diagnosis.

Therefore, our main goal was to explore the self-reported prevalence of psoriasis in the axSpA population using a questionnaire with prototypical color pictures and to compare this with the prevalence of a verified diagnosis. Secondly, to explore if there are differences in disease and patient characteristics between axSpA patients with and without self-reported psoriasis including with and without a verified psoriasis diagnosis.

## Patients and methods

### Study cohort

All patients from the Groningen Leeuwarden Axial Spondyloartritis (GLAS) cohort included before June 2016, were approached for this study. The GLAS cohort is an on-going, longitudinal, prospective observational cohort study which started in 2004. Clinical data and patient-reported outcomes are collected during standardized out-patient follow-up visits every 6 to 12 months. For the present study, data from the cohort database were used as described previously [11]. The GLAS cohort was approved by the local ethics committees of the Medical Center Leeuwarden (MCL) and the University Medical Center Groningen (UMCG). All patients provided written informed consent according to the Declaration of Helsinki, in which they also gave written permission to be approached (non-committal) for SpA-related research. No additional informed consent was needed for this study according to the local ethical committee.

### Psoriasis questionnaire and verification of diagnosis

Patients were asked to fill in a questionnaire about several skin diseases in axSpA and return it by post using a stamped return envelope. After 3 weeks, a reminder was sent to the non-respondents. Anonymous returns and largely incomplete answered questionnaires were excluded.

The applied diagnostic psoriasis question “Do you have psoriasis?” was supported with the following brief elucidation: “Psoriasis is a skin disease in which red and scaly patches develop on the body, especially on the elbows, knees, and hairy head. The nails can also be affected, and sometimes there are joint complaints.” In addition, to enable patients to self-assess the presence of psoriasis more accurately, prototypical color pictures of psoriasis lesions were added. Patients could reply with “no,” “yes, in the past,” or “yes, currently.” Both “yes, in the past” and “yes, currently” were considered self-reported symptoms. Subsequently, patients were asked whether they had received a diagnosis, and if so, who made the diagnosis.

In patients who indicated having psoriasis symptoms, a medical record check was done to verify previously diagnosed psoriasis. The diagnosis of psoriasis was considered valid if it was described in the medical record by a dermatologist or rheumatologist.

For assessing the severity of psoriasis, a schematic image of body surface area was used, answer options were “mild,” “moderate,” “severe,” or “not currently active.” “Active” psoriasis was determined by those who selected “yes, currently” to the first question minus those who reported “yes, currently not active” to the severity question. In an open-ended question, patients were asked to name the location of the psoriasis skin lesions.

### Statistical analysis

The prevalence rate of patient-reported psoriasis according to the questionnaires and verified psoriasis diagnosis according to the clinical record were calculated. Descriptive statistics were applied for all relevant variables; results were expressed as number of patients (%), mean ± standard deviation (SD), or median (interquartile range [IQR]) for categorical, normally distributed, and non-normally distributed data, respectively. Group comparison for patients with versus without self-reported symptoms of psoriasis was done using the chi-square test or Fisher’s exact test for categorical variables and Independent Sample *t*-tests or Mann–Whitney *U* tests for continuous variables. *P*-values ≤ 0.05 were considered as statistically significant. Statistics were performed using IBM SPSS 23.0 software for Windows (SPSS, Chicago, IL, USA).

## Results

### Questionnaire response and patient characteristics

**Fig. 1:**
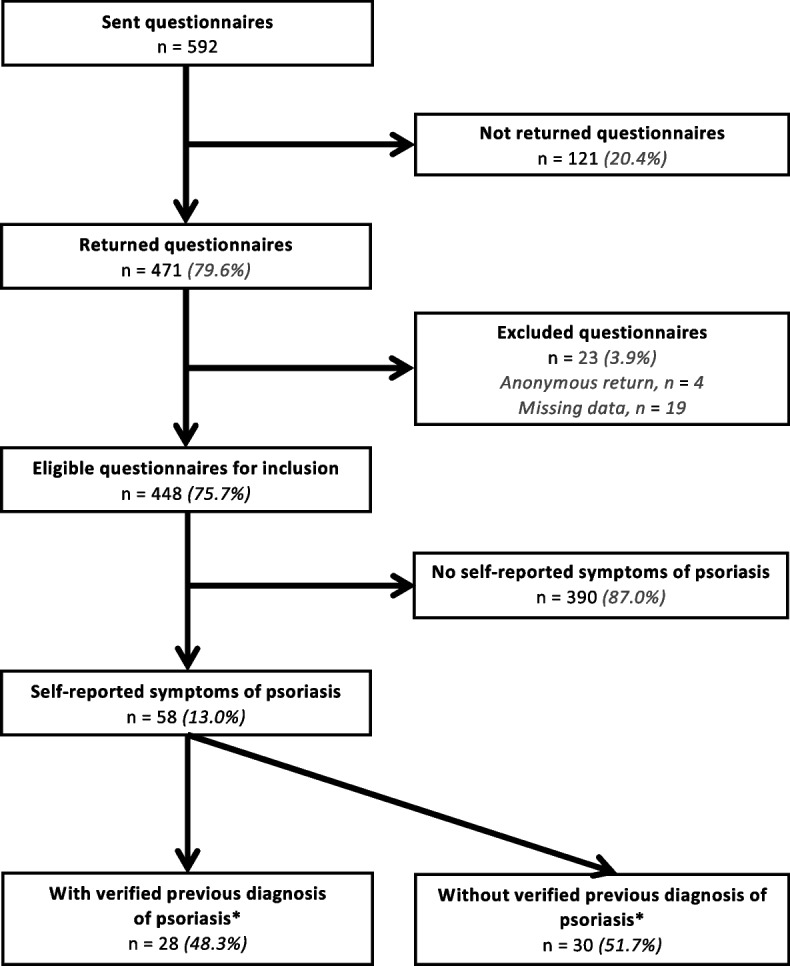
Questionnaire response and self-reported psoriasis symptoms and verified diagnosis. Asterisk (*) symbol indicates the following: Statement of confirmed psoriasis diagnosis made by dermatologist or rheumatologist in patient medical record

Of the 592 questionnaires sent, 471 (79.6%) were returned, of which 448 (75.7%) were eligible for analysis (Fig. 1). Of these, 341 (76.1%) were diagnosed with AS, 90 (20.0%) with nr-axSpA and for 17 (3.8%) patients sub-classification data was missing. Mean ± SD age of the 448 included patients was 50.1 ± 12.7 years, 64.1% were male, median [IQR] SpA symptom duration was 21.0 [12.0–32.0] years, 78.5% were HLA-B27 positive and mean Ankylosing Spondylitis Disease Activity Score (ASDAS_CRP_) was 2.2 ± 1.0 (Table 1). The male–female ratio between the 448 included and 147 excluded patients was similar. The excluded patients were significantly younger (43.3 ± 13.6, *p* < 0.001). All other patent axSpA patient characteristics were similar in both groups.

**Table 1:** AxSpA characteristics of patients with and without self-reported psoriasis symptoms and with and without verified psoriasis diagnosis

	Total included axSpA patients (*n* = 448)	AxSpA patients **without** self-reported psoriasis (*n* = 390)	AxSpA patients **with** self-reported psoriasis (*n* = 58)	AxSpA patients with self-reported psoriasis **with** verified diagnosis (*n* = 28)	AxSpA patients with self-reported psoriasis **without** verified diagnosis (*n* = 30)
Age (years)	52.2 ± 12.8	50.1 ± 12.7	52.2 ± 12.8	49.3 ± 13.0	55.0 ± 12.2
Sex (% male)	287 (64.1)	241 (62.3)	44 (75.9)*	19 (67.9)	25 (83.3)
HLA-B27 +	339 (78.5)	294 (78.6)	43 (74.1)	19 (73.1)	24 (82.8)
Smoking	289 (67.4)	248 (67.0)	38 (65.5)	18 (69.2)	20 (66.7)
BMI, at baseline (kg/m^2^)	26.3 [23.4–29.1]	26.1 [23.2–29.1]	27.1 [25.3–30.1]*	27.9 [26.2–30.1]	26.6 [25.2–29.5]
Duration of SpA symptoms^a^ (years)	21.0 [12.0–32.0]	22.0 [12.0–32.8]	19.0 [9.0–33.0]	17.0 [6.0–29.0]	24.0 [14.0–41.0]
Age of onset SpA symptoms^a^ (years)	24.9 [19.5–33.1]	24.5 [19.0–32.3]	26.8 [21.3–37.1]	29.5 [23.3–35.5]	25.7 [20.0–37.9]
Anti-TNF history	252 (56.3)	212 (54.8)	37 (63.8)	18 (64.3)	19 (63.3)
History of IBD	46 (10.3)	34 (8.8)	11 (19.0)*	4 (14.3)	7 (23.3)
History of peripheral arthritis	161 (38.6)	134 (37.4)	25 (43.1)	11 (42.3)	14 (46.7)
History of uveitis^a^	134 (29.9)	118 (30.5)	17 (29.3)	4 (14.3)	4 (13.3)
History of enthesitis	89 (20.0)	79 (20.6)	8 (13.8)	5 (17.9)	10 (33.3)
History of dactylitis	30 (6.8)	24 (6.2)	6 (10.3)	4 (14.3)	2 (6.7)
ASDAS_CRP_	2.2 ± 1.0	2.2 ± 1.0	2.0 ± 1.0	1.9 ± 0.9	2.1 ± 1.0
BASDAI	3.7 ± 2.2	3.7 ± 2.2	3.7 ± 2.2	3.5 ± 2.2	3.8 ± 2.2
CRP	3 [2–6]	3 [2–6]	2.0 [1.0–5.0]*	2.0 [1.2–3.3]	2.0 [1.0–6.1]
ASQoL	4.5 [1–9]	4.4 [1–9]	5.6 [1.3–9.0]	3.8 [1.8–9.3]	7.0 [1.8–8.6]

Values are presented as the number of patients (%), mean ± SD or median [interquartile range]

^*^Comparison is significant, *p*-value (< 0.05)

^a^7.5–10% missing data

### Prevalence of axSpA patient self-reported psoriasis symptoms and verified diagnosis

Of the 448 respondents, 58 (13.0%) had a positive self-screening for psoriasis symptoms, currently or in the past. In 28 (48.3%) patients, psoriasis diagnosis could be verified by a medical record check. This results in a psoriasis prevalence rate between 6.3% (28/448) and 13.0% (58/448) within the GLAS cohort. In the other 30 (51.7%) patients with a positive self-screening for psoriasis, no previous verified psoriasis diagnosis could be confirmed by medical record check (Fig. 1).

### Patient-reported severity and location of psoriasis symptoms

Psoriasis symptoms, present at the time of the questionnaire response date, were reported by 31 of 58 (53%) patients. Mild psoriasis symptoms were reported by 23 (74.2%), moderate by 6 (19.4%), and severe by 2 (6.5%) patients. Legs (44%), head (41%), and arms (33%) were the most frequently self-reported affected body sites. In 14 out of these 31 patients (45%), a diagnosis could not be verified by checking clinical records. Of these, all but one (93%) reported mild psoriasis symptoms. Only the remaining patient reported moderate to severe psoriasis. Head and arms were the most frequently affected body sites among the 14 patients without a verified diagnosis, which was reported by 4 (29%) and 6 (43%) patients respectively.

### Differences in patient characteristics

Table 1 shows that axSpA patients with self-reported psoriasis symptoms were more often male and had more often a history of IBD than patients without self-reported psoriasis symptoms (76% vs. 62% and 19% vs. 8.8% resp., *p* < 0.05). Furthermore, BMI was slightly but significantly higher in the axSpA patients with self-reported psoriasis (27.1 [25.3–30.1] vs. 26.1 [23.2–29.1], *p* < 0.05). The 28 patients with self-reported psoriasis (currently or in the past) and a verified diagnosis (medical record check) also reported significantly more moderate to severe psoriasis symptoms than the 30 patients with a positive self-screening and no verified diagnosis (25% vs. 3.3%, *p* < 0.05). Psoriasis lesions located on the torso area, intergluteal cleft and legs were reported more often by the 28 patients with a confirmed diagnosis (18% vs. 3.3%, 25% vs. 0%, and 43% vs. 7%, resp., *p* < 0.05).

## Discussion

In this cross-sectional study, the self-reported prevalence of psoriasis using a questionnaire with prototypical pictures of psoriasis was investigated in a multicenter Dutch axSpA cohort in daily clinical practice. In total, 13% (58/448) of axSpA patients reported to have psoriasis symptoms, currently or in the past. In only about half of these (*n* = 28), patients’ diagnosis was confirmed by a dermatologist or rheumatologist prior to this study, indicating an overall cohort prevalence of psoriasis 6.3%, which presumably means that psoriasis might have been underdiagnosed in this population.

To the best of our knowledge, there is no previous publication concerning the self-reporting prevalence of psoriasis symptoms with prototypical pictures in axSpA. One review from 2015 showed that the pooled prevalence of psoriasis diagnosis, either self-reported or in medical record by screening or interview, in patients with AS was 9.3% (95% confidence interval (CI) = 8.1–10.6%) [1]. This is somewhat higher compared to the verified psoriasis prevalence rate in the current study, which indicates the potential value of this self-screening questionnaire tool, creating awareness of present psoriasis in axSpA patients which will help physicians to earlier recognize and diagnose psoriasis. Importantly, in our study, most patients with currently active psoriasis symptoms but *without* a verified diagnosis reported only mild symptoms which may have contributed to the relatively low prevalence rate. In addition, a physician may not appreciate the full burden of disease and/or patients will not mention skin symptoms unless specifically asked for [12]. This may especially occur in the case of psoriasis symptoms in the past and/or when having mild psoriasis symptoms. The head was one of the most prevalent psoriasis locations on the body indicated by patients reporting psoriasis without verified diagnosis. In axSpA, plaques psoriasis is most often reported therefore psoriasis located on the head may have been missed more easily by the rheumatologists [4]. Previous longitudinal analyses of axSpA patients within the OASIS cohort and our GLAS cohort showed associations of diagnosed ESM including psoriasis with worse QoL, which underlines the clinical relevance of not missing the psoriasis diagnosis.

Previously, a validation study of the self-reporting question “Have you had or do you have psoriasis” in a general Norwegian population and found an estimated sensitivity of 56% (95% CI = 44–68%), specificity of 99% (95% CI = 98–99%), positive predictive value of 78% (95% CI = 69–85%), and negative predictive value of 96% (95% CI = 94–98%) using skin examination by dermatologist in case of active psoriasis symptoms and medical record or previous skin biopsy verification in case of currently inactive psoriasis as golden standard. The positive predictive value increased from 78 to 84% if the psoriasis question was combined with the additional question “Have you been diagnosed with psoriasis by a dermatologist?” [13]. We believe that adding a brief informative description and especially prototypical color pictures of different types of psoriasis lesions (as used in the current study) will contribute to even higher validation outcomes [14, 15].

Interestingly, patients with self-reported psoriasis were more often male than patients without self-reported psoriasis. One previous study in the general population also reported that in patients with undiagnosed psoriasis, there was a trend towards male sex [12].

Another notable result is that a history of IBD was more common among patients with self-reported psoriasis. AxSpA, IBD, and psoriasis are all so-called immune-mediated inflammatory diseases (IMID). IMID is a concept of a group of prevalent diseases that share an imbalance in inflammatory pathways central to their pathogenesis. Multiple studies have indicated that multiple IMID may co-exist in the same patient, which is not inexplicable regarding the theory of a shared deregulation of certain inflammatory pathways [16].

Strengths of this study are the well-defined cohort with a large amount of standardized data and a high questionnaire response rate in comparison to other studies. A limitation of this study is that it was restricted to information from questionnaires, however, verified by medical records. To determine the clinical value of our psoriasis screening questionnaire with prototypical questions a validation study including clinical consultation by a dermatologist is needed.

In conclusion, this study indicates that a self-reported questionnaire with prototypical pictures of psoriasis symptoms could be beneficial in tracing axSpA patients with undiagnosed psoriasis. We recommend that physicians as well as axSpA patients should be made aware of (developing) psoriasis during the course of their disease.

## Data Availability

The datasets used and/or analyzed during the current study are available from the corresponding author on reasonable request.

## Abbreviations

ESM: Extra-skeletal manifestations
axSpA: Axial spondyloarthritis
ASAS: Assessment of Spondyloarthritis International Society
IBD: Inflammatory bowel diseases
AS: Ankylosing spondylitis
nr-axSpA: Non- radiographic axial spondyloarthritis
SpA: Spondyloarthritis
IL: Interleukin
TNFα: Tumor necrosis factor alpha
GLAS: Groningen Leeuwarden Axial Spondyloarthritis cohort
MCL: Medical Center Leeuwarden
UMCG: University Medical Center Groningen
SD: Standard deviation
IQR: Interquartile range
ASDAS_CRP_: Ankylosing Spondylitis Disease Activity Score
CI: Confidence interval
IMID: Immune-mediated inflammatory diseases
